# Virologic suppression in response to antiretroviral therapy despite extensive resistance within HIV-1 reverse transcriptase after the first virologic failure

**DOI:** 10.1186/s12879-018-3400-6

**Published:** 2018-10-12

**Authors:** Marta Iglis Oliveira, Valter Romão de Souza Junior, Claudia Fernanda de Lacerda Vidal, Paulo Sérgio Ramos de Araújo

**Affiliations:** 1 Programa de Pós-graduação em Ciências da Saúde, Universidade Federal de Pernambuco, Av. Prof. Moraes Rego 1235, Recife, 50670-901 Brazil; 2 Instituto Aggeu Magalhaes, FIOCRUZ, Av. Prof. Moraes Rego 1235, Recife, 50670-901 Brazil; 3 Faculdade de Medicina do Recife, Universidade Federal de Pernambuco, Av. Prof. Moraes Rego, 1235, Recife, Pernambuco Brazil

**Keywords:** HIV-1 drug resistance, Genetic diversity, Subtypes, Antiretroviral therapy

## Abstract

**Background:**

Incomplete virologic suppression results in mutations associated with resistance and is a major obstacle to disease control. We analyzed the genotypic profiles of HIV-1 patients at the time of the first virologic failure and the response to a salvage regimen after 48 weeks.

**Methods:**

This work was a cross-sectional, retrospective, analytical study based on data collected from medical records and genotyping tests between 2006 and 2016. The sample consisted of data on individuals living with HIV (PLWH) from three major reference centers.

**Results:**

A total of 184 patients were included in the data analysis. Viral subtype B was the most common (81.3%) as well as M184 V/I (85.3%) and K103 codon mutations (65.8%). Forty-eight weeks after switching to a salvage regimen, 67.3% of patients achieved an undetectable viral load.

**Discussion:**

The number of mutations associated with nucleos(t)ide reverse transcriptase inhibitors (NRTI(t)s) did not affect virologic suppression (9.3% for zero NRTI(t)-associated mutations vs 48.6% for 1–2 NRTI(t)-associated mutations vs 42.1% for ≥3 NRTI(t)-associated mutations, *p* = 0.179). An ARV time (the beginning of the first ARV regimen up to genotyping) of > 36 months was a protective factor for detectable viral load (PR = 0.60, 95% CI = 0.39–0.92, *p* = 0.020) and a risk factor for developing ≥3 NRTI(t)-associated mutations (PR = 2.43, 95% CI 1.38–4.28, *p* = 0.002).

**Conclusions:**

We found that extensive resistance to NRTI(t)s at the time of the first virologic failure did not impact virologic suppression at 48 weeks after switching to a second-line therapy based on NRTI(t)s plus protease inhibitors.

## Background

Approximately 21 million people are living with HIV (PLHIV) and receiving antiretroviral therapy [1], which is responsible for a significant decrease in their morbidity and mortality as well as in the risk of transmission [2, 3]. However, the importance of this therapy to global health has been threatened by an increased prevalence of resistance to antiretrovirals (ARVs), which has increased from 11 to 29% since 2001 [4]. Of the individuals under ARV treatment, 20% will have to switch ARVs due to virologic failure [5, 6]. Thus, drug resistance is a real obstacle to viral suppression and disease control [1].

In Brazil, approximately 60% of PLHIV receive ARVs [7] through a program by the Brazilian Ministry of Health. From 2004 to 2017, the first-line therapy in the country was based on regimens involving efavirenz (EFV), a non-nucleoside reverse transcriptase inhibitor (NNRTI). In 2013, tenofovir (TDF) became the preferred nucleos(t)ide reverse transcriptase inhibitor (NRTI(t)), and it was given in a single dose combined with lamivudine (3TC) and EFV [8]. In 2017, dolutegravir (DTG), an integrase inhibitor that was administered with TDF/3TC, became the first-line therapy [9].

Genotypic and immunovirologic data from patients following the first virologic failure are scarce in Brazil, and they are limited to certain regions of the country. A recent study from the city of São Paulo^10^ on patients who failed first-line therapy between 2013 and 2015 found a higher prevalence of the M184 V/I mutation (74.3%) and K103 codon (56.7%), similar to that found in other Brazilian states [11]. Among the 205 subjects who started the second-line therapy with NRTI(t)s combined with protease inhibitor/ritonavir (PI/r), 76.6% experienced virologic suppression (< 200 copies/ml), despite the extensive resistance of the virus to NRTI(t)s [10].

Low- and middle-income countries in sub-Saharan Africa, where 70% of PLHIV live [1], access 2 NRTI(t) + NNRTI as a first-line therapy [12]. This regimen has a low genetic barrier and easily results in cross-resistance. Thus, the sensitivity of NRTI(t)s may be compromised by multiple mutations that arise during the first virologic failure. In addition, NRTI(t)s have recognized risks of toxic effects. These factors are concerning and have led to the decision to retain this drug class as a second-line therapy, and they have motivated the development of randomized clinical trials to evaluate the efficacy of the salvage regimen with NRTI(t)s plus PI/r.

Mutations are associated with high reduced susceptibility or virologic response to relevant NRTIs. Mutations reduce NRTI susceptibility or virologic response, which contributes to reducing susceptibility in combination with other NRTI-resistant mutations. The impact of mutations associated with NRTI(t)s during the second-line regimen was evaluated through the SECOND-LINE, EARNEST and SELECT studies [13–15] and they were recently reevaluated in a meta-analysis [20], which showed the success of salvage regimens based on NRTI(t)s combined with PI/r. In this study, we analyzed the genotypic profile of HIV-1 in patients during the first virologic failure and the predictors of virologic success 48 weeks after switching to a salvage regimen.

## Methods

This work was a cross-sectional, retrospective, multicenter study evaluating genotypic resistance tests of PLHIV during the first virologic failure in the city of Recife, the capital of the state of Pernambuco, an important medical center in Northeast Brazil. The patients were enrolled in three large centers specializing in the care of PLHIV. We reviewed tests that were performed from January 2006 to December 2016 and included patients over 18 years of age who had used ARVs for more than six months and presented two consecutive viral load measurements, with an interval of at least 30 days, and values greater than 1000 copies/ml. Individuals who underwent ARV switching due to virologic failure without genotyping tests, those who used ARVs before receiving the first-line therapy, those showing an absence of mutations associated with resistance in genotyping, and those with insufficient data were excluded.

The immunovirologic and genotypic data and the history of ARV use were collected from genotyping tests, medical charts and through the Laboratory Test Control System (Sistema de Controle de Exames Laboratoriais - SISCEL- online platform). To identify the mutations associated with resistance, the ViroSeqTM HIV-1 Genotyping System kit (Celera Diagnostics, Alameda, CA, USA) was used from 2006 to 2008 and the TRUGENE System (Siemens, Munich, Germany) was used from 2009 to 2016 by the laboratories that served the National Genotyping Network (Rede Nacional de Genotipagem - Renageno). The viral load (VL) was measured until July 2013 using the Versant HIV-1 RNA 3.0 assay (bDNA, Siemens, USA); real-time qPCR was used after this date (Abbott Laboratories, USA). The CD4 T-cell counts (CD4) were performed by flow cytometry (BD, USA).

The Stanford HIVdb program version 8.4 (https://hivdb.stanford.edu/hivdb/by-mutations) was used to interpret the genotype resistance. The mutations were analyzed individually using a list provided by the International AIDS Society (IAS) in 2017, [16] and the Stanford HIVdb program and categorized as mutations associated with NRTI(t), NNRTI and major and accessory mutations for protease inhibitors (PI). Forty-eight weeks after switching to second-line therapy, virologic suppression was considered successful when an undetectable viral load was found by the given method. Viral load values made available 60 days before or after the date of completion (for the 48 weeks) were considered. The time on ARVs was the period between the beginning of the first ARV regimen and the performance of the genotyping test. The time in virologic failure was the period between virologic failure detection (two consecutive viral loads above 1000 copies/ml) and the switch to the salvage regimen.

The genotypic sensitivity score (GSS) was calculated individually only for the drugs used by each patient in the salvage regimen. A value of 1 was attributed to drugs with full activity, 0.75 for a low potential resistance level, 0.5 for a resistance level, 0.25 for an intermediate resistance level and zero if the drug showed a lack of activity.

SPSS 13.0 (Statistical Package for the Social Sciences) for Windows and Excel 2010 were used for the statistical analysis. The results are presented in the tables with their respective absolute and relative frequencies. The mean or median values for the quantitative variables were calculated according to the normality of their distribution. The normality of the quantitative variables was tested using the Kolmogorov-Smirnov test.

We tested for the presence of an association using the chi-square test and Fisher’s exact test for categorical variables. To identify the predictive factors that independently influenced virologic suppression and the number of mutations associated with NRTI(t)s, we used the STATA/SE 12.0 programs. For the multivariate analysis, the Poisson regression model was used, taking into account the variables that obtained significance ≤0.20 in the bivariate analysis. The prevalence ratio was calculated with a 95% confidence interval. A *p* < 0.05 was considered statistically significant. Poison-regression was chosen as excellent alternative to estimate the adjusted prevalence ratio for confounding variables in cross-sectional studies compared to Cox and log-binominal logistic regression. The study was approved by the institutional ethics committee (CEP-CCS-UFPE/1.985.922).

## Results

A total of 534 medical records were evaluated, of which 270 were excluded due to a lack of genotyping tests when patients switched ARVs after the first virologic failure. In 59 medical records, there was no selective pressure during genotyping. Therefore, the absence of mutation does not represent reality. Thus, 59 patients were excluded. Non-adherence to ARV use at the time of genotyping was recorded in the medical charts of the 59 excluded patients with absence of resistance-associated mutations. Furthermore, 21 patients were not either illegible or had insufficient data.

A total of 184 patients were included in the first virologic failure for analysis. The majority of the participants were male (73.4%) and the mean age at virologic failure was 41 (±9.4) years. The median CD4 (*n* = 155) and VL (*n* = 141) pre-ARV were 176 cells/m3 and 130,000 copies/ml, respectively.

This sample is representative considering the limited resources scenario. It represents a decade of genotyping in the first virologic failure in Pernambuco, Northeast Brazil. In 2016, we were able to access genotyping easily. Between 2006 and 2016, there was therapeutic rescue without genotyping due to the logistic difficulty in this region of Brazil. Therefore, more than 50% of the sample was excluded by changing the scheme without genotyping.

### Factors associated with mutations, immunovirologic and ARV characteristics at the time of genotyping

The genotypic, immunovirologic and ARV characteristics at the time of genotyping are described in Table 1. The NRTI(t)-associated mutation M184 V/I emerged in 78.4% of the subtype B sequences and in 21.6% of the non-B subtype sequences (*p* = 0.009). Most individuals (89.7%) exhibited at least one mutation for NRTI(t). The association of the presence of ≥3 NRTI(t)-associated mutations with CD4 pre-ARVs < 200 cell/mm3 (66.7% vs 33.3% for CD4 pre-ARVs ≥200, prevalence ratio [PR] = 1.52, CI = 1.00–2.31, *p* = 0.041), with CD4 at the time of genotyping < 200 cells/mm3 (59.4% vs 23.4% for CD4 200–350 vs 17.2% for CD4 > 350, PR = 2.04, CI = 1.18–3.55, *p* < 0.011), and with a time on ARV of greater than 36 months (81.2% vs 18.8% for Δt < 36 months, *p* = 0.003) were significant, according to Table 2. In addition, a VL of 10,000–100,000 copies/ml was associated with the highest number of NRTI(t)-associated mutations (Table 2).

**Table 1:** Demographic and clinical characteristics of study participants

Variables	*N*	%
Age (years) (mean ± SD)	41,1 ± 9,4	
Gender		
Male	135	73,4
Female	49	26,6
Baseline CD4 count, median (Q1; Q3)	234, 0 (118, 0; 361, 0)	
CD4		
< 200	78	42,4
200 a 350	50	27,2
> 350	47	25,5
Data unavailable	9	4,9
VL (copies/ml), median (Q1; Q3)	19.812,5 (7.036,0; 78.674,2)	
VL (copies/ml)		
≤ 10.000	59	32,1
> 10.000 a 100.000	75	40,7
> 100.000	37	20,1
Data unavailable	13	7,1
∆t on ART (months), median (Q1;Q3)	54, 5 (29, 0; 90, 0)	
Δt in virologic failure (months), median (Q1;Q3)	17, 0 (10, 0; 34, 0)	
NRTI in failing regimen	184	100,0
AZT/3TC	126	68,5
TDF/3TC	48	26,1
Others	10	5,4
NNRTI in failing regimen	155	84,2
EFV	135	87,1
NVP	20	12,9
PI in failing regimen	29	15,8
LPV/r	16	55,2
ATV/r ou ATV 400 mg	12	41,4
Outros	1	3,4
Viral Subtype	171	92,9
B	139	81,3
F	31	18,1
BF	1	0,4

*VL* = viral load, *ART* antiretroviral therapy, *NNRTIs* nonnucleoside reverse transcriptase inhibitors, *NRTIs* nucleoside/nucleotide reverse transcriptase inhibitors, *AZT* zidovudine; *3TC* lamivudine, *TDF* tenofovir, *EFV*: efavirenz, *NVP* nevirapine, *PI* protease inhibitor; *LPV/r* lopinavir/ritonavir, *ATV/r* atazanavir/ritonavir

**Table 2:** Risk factors associated with HIV-1 drug resistance

Number of resistance mutations					
Factors	≥ 3 *n* (%)	< 3 *n* (%)	PR	PR 95% IC	*P*-value
Age (years)					0, 806^a^
18–30	7 (10, 1)	11 (11, 5)	0, 90	0, 49 – 1, 66	
31–50	52 (75, 4)	68 (70, 8)	1, 00	–	
> 50	10 (14, 5)	17 (17, 7)	0, 85	0, 50 – 1, 46	
Gender					0, 476^a^
Male	53 (76, 8)	69 (71, 9)	1, 17	0, 75 – 1, 81	
Female	16 (23, 2)	27 (28, 1)	1, 00	–	
Viral subtype					0, 771^a^
B	50 (80, 6)	74 (78, 7)	1, 00	–	
No-B	12 (19, 4)	20 (21, 3)	0, 93	0, 57 – 1, 53	
VL before ART (copies/ml)					0, 570^b^
≤ 10.000	4 (7, 5)	3 (4, 3)	1, 00	–	
> 10.000 a 100.000	17 (32, 1)	27 (38, 6)	0, 68	0, 32 – 1, 42	
> 100.000	32 (60, 4)	40 (57, 1)	0, 78	0, 39 – 1, 55	
CD4 before ART					0, 041^a^
< 200	40 (66, 7)	39 (49, 4)	1, 52	1, 00 – 2, 31	
≥ 200	20 (33, 3)	40 (50, 6)	1,00	–	
VL in virologic failure (copies/ml)					0, 042^a^
≤ 10.000	15 (24, 6)	38 (71, 7)	1, 00	–	
> 10.000 a 100.000	34 (55, 7)	33 (36, 3)	1, 79	1,10 – 2, 92	
> 100.000	12 (19, 7)	20 (22, 0)	1,33	0,71 – 2, 46	
CD4 in virologic failure					0, 011^a^
< 200	38 (59, 4)	33 (35, 9)	2, 04	1,18 – 3, 55	
200–350	15 (23, 4)	28 (30, 4)	1, 33	0, 69 – 2, 56	
> 350	11 (17, 2)	31 (33, 7)	1, 00	–	
ART regímen at NRTI baseline					0, 189^a^
TDF	12 (17, 4)	25 (26, 0)	1, 00	–	
No-TDF	57 (82, 6)	71 (74, 0)	1, 37	0, 83 – 2, 27	
Time to virologic failure (months)					0, 221^a^
< 12	13 (19, 4)	28 (30, 4)	1, 00	–	
12–24	25 (37, 3)	34 (37, 0)	1,3 4	0, 78 – 2, 29	
> 24	29 (43, 3)	30 (32, 6)	1, 55	0, 92 – 2, 60	
Time on ART (months)					0, 003^a^
≤ 36	13 (18, 8)	39 (40, 6)	1, 00	–	
> 36	56 (81, 2)	57 (59, 4)	1, 98	1, 19 – 3, 29	

*ART* antiretroviral therapy, *PR* prevalence ratio, *CI* confidence interval, *VL* viral load, *cell T CD4* CD4 T cell count, *TDF* tenofovir, *NRTIs* nucleoside/nucleotide reverse transcriptase inhibitors

^a^Chi-square test

^b^Fisher’s exact test

After analysis using the Poisson regression model (Table 3), only patients who were on ARVs for more than 36 months until genotyping (PR = 2.43, 95% CI = 1.38–4.28, *p* = 0.002) and who developed VL at the time of genotyping of 10,000–100,000 copies/ml (PR = 2.09 95% CI = 1.25–3.50, *p* = 0.005) and > 100,000 copies/ml (PR = 2.20, 95% CI = 1.14–4.27, p = 0.005) had a higher risk of having ≥3 NRTI (t)-associated mutations.

**Table 3:** Analysis using the poisson regression model

Variables	PR	PR 95% CI	*P*-value
CD4 before ARTs (cells/mm^3^)			
< 200	1, 46	0, 95 – 2, 24	0, 084
≥ 200	1, 00	–	
HIV AIDS (Auckl)			
≥ 10.000	1, 00	–	
> 10.000 a 100.000	2, 09	1, 25 – 3, 50	0, 005
> 100.000	2, 20	1, 14 – 4, 27	0, 018
Time to failure (months)			
≤ 36	1, 00	–	0, 002
> 36	2, 43	1, 38 – 4, 28	

*PR* prevalence ratio, *CI* confidence interval, *CD4 CD4* + T cell count, *VL* viral load

**Fig. 1:**
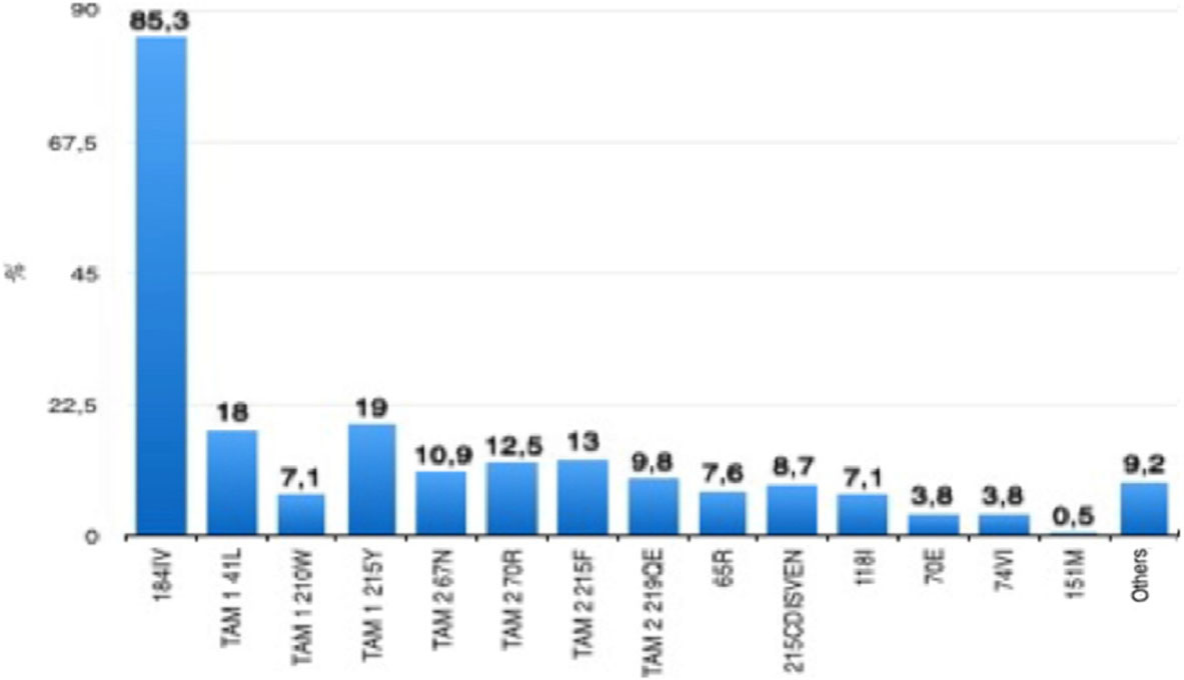
The prevalence of drug resistance to NRTI among 184 HIV-1 infected patients with virologic failure

There was a trend towards the emergence of three or more thymidine-associated mutations (TAMs) when the time on ARVs was greater than 36 months (92% vs 8% for Δt on ARVs ≤36 months, *p* = 0.051). K65R emerged in 7.6% of 184 genotyping tests (Fig. 1) and was observed in 27.1% of patients who were exposed to TDF/3TC. Mutations in the K103 codon were observed in 65.8% (*n* = 121/184) of the genotyping tests. Among those who used NNRTI, this mutation occurred in 78.0% (*n* = 121/155). The NNRTI-associated mutation 90IV was more frequent in the non-B subtype (57.1% vs 42.9% for subtype B, *p* = 0.024) and the 190AS was most prevalent for etravirine.

Considering the 29 patients who used PI, 55.2% (*n* = 16) were on lopinavir/ritonavir (LPV/r) and 41.4% (*n* = 12) were on atazanavir (ATV) (Table 1). The PI-associated major and accessory mutations were 82AF (51.7%) and 10IFV (62%), respectively.

The PI-associated major mutation 54VL emerged frequently in subtype B sequences (58.3% vs 41.7% for non-B subtype, *p* = 0.05), as did the PI-associated accessory mutations 10IFV (69% vs 31% for non-B subtype, *p* = 0.019) and 20RT (60% vs 40% for non-B subtype, *p* = 0.010).

The total number of mutations was significantly equal to or greater than seven among the users who had a CD4 count below 200 cells/m3 at the time of genotyping (52.9% vs 27.6% for CD4 200–350 cells/m3 vs 19.5% for CD4 > 350 cells/m3, *p* = 0.045).

### Salvage therapy characteristics

There was a loss of follow-up in eight (4.3%) patients after the genotyping test was performed. Thus, 176 (95.7%) patients switched to the salvage regimen. Of these individuals, 109 (61.9%) patients used TDF/3TC as the NRTI(t), followed by 28 (15.9%) who used TDF/AZT(zidovudine)/3TC and 27 (15.3%) AZT/3TC. Only nine (5.1%) individuals did not have NRTI(t)s included in their salvage regimen.

PIs were present in the salvage regimen of 99.4% of the individuals; only one patient did not use PI. LPV/r was the most frequently prescribed (48.5%) followed by ATV/r (33.1%). Integrase inhibitors made up the salvage regimen of 35 (19.9%) individuals. Raltegravir (RAL) was the most frequently used one (*n* = 32). When the salvage regimen consisted of two active drugs (GSS = 2), 79.5% of this population showed genotyping with less than three NRTI(t)-associated mutations (79.5% vs 20.5% for ≥3 NRTI(t)-associated mutations, *p* < 0.000).

The susceptibility of etravirine was not associated with the use of EFV or nevirapine (NVP) or with the number of NNRTI-associated mutations. This treatment remained fully active in 63.2% of the sequences among the patients who experienced a failed NNRTI therapy. We found a similar susceptibility of AZT and TDF (64.2% vs 65.9%). Among the PI/r users, darunavir/ritonavir (DRV/r) showed the highest percentage of sensitivity (69%), and ATV/r experienced the highest percentage of resistance (79.3%).

Most of the salvage regimens showed GSS equal to two (42.6%) or greater than two (44.3%). We found that in most of the regimens with a GSS greater than 2, the patient was in virologic failure for more than 24 months (46% vs 30.8% for Δt in virologic failure 12–24 months vs 23.2% for Δt in virological failure < 12 months, *p* = 0.013) and on ARV for more than 36 months (76.9% vs 23.1% for Δt in ARV 36 months, *p* = 0.004).

When virologic failure occurred during LPV/r use, the GSS of the salvage therapy regimen was significantly higher than two (85.7% vs 14.3% for GSS = 2 vs 0% for GSS < 2, *p* = 0.001). The significant majority of TDF users in virologic failure were salvaged with an ARV regimen with a GSS equal to two (62.2% vs 27.0% for GSS > 2 vs 10.8% for GSS < 2, *p* = 0.023).

### Predictors of undetectable viral loads and CD4 T-cell count variation at 48 weeks after the onset of the salvage regimen

Of the 176 patients who received documented salvage therapy prescriptions, we were able to obtain the VL value 48 weeks after switching ARVs for 159 (90.3%). Of these, 67.3% (*n* = 107) experienced virologic suppression and 11.3% did not reach an undetectable VL but had below 400 copies/ml. The majority of the population with undetectable VL had more than 36 months of ARV use until genotyping (73.8% vs 26.2% for 36 months, *p* = 0.022), and 42.0% were in virologic failure for 12 to 24 months (42.0% vs 34.6% for > 24 months vs 23.4% for < 12 months, *p* = 0.029) (Table 4).

**Table 4:** Characteristics related to the irrigation regimen, ART time and virological failure time associated with virological suppression after 48 weeks from the start of the rescue scheme

Viral load (VL)					
Characteristics	Detectable *n* (%)	Undetectable *n* (%)	PR	PR 95% IC	*P*-value
Time to virologic failure (months)					0, 029^a^
< 12	22 (42, 3)	25 (23, 4)	1, 00	–	
12–24	13 (25, 0)	45 (42, 0)	0, 48	0, 27 – 0, 84	
> 24	17 (32, 7)	37 (34, 6)	0, 67	0, 41 – 1, 11	
∆t on ART (months)					0, 022^a^
≤ 36	23 (44, 2)	28 (26, 2)	1, 00	–	
> 36	29 (55, 8)	79 (73, 8)	0, 60	0, 39 – 0, 92	
ART rescue NRTI					0, 711^b^
TDF/3TC	36 (69, 2)	62 (57, 9)	1, 00	–	
AZT/3TC	7 (13, 5)	18 (16, 8)	0, 76	0, 39 – 1, 50	
TDF/AZT/3TC	7 (13, 5)	19 (17, 8)	0, 73	0, 37 – 1, 45	
DDI/3TC	0 (0, 0)	1 (0, 9)	–	–	
ABC/3TC	1 (1, 9)	1 (0, 9)	1, 36	0, 33 – 5, 58	
NNRTI	1 (1, 9)	6 (5, 6)	0, 39	0, 00 – 2, 43	
ART rescue PI					0, 013^b^
LPV/r	24 (46, 1)	55 (51, 5)	2, 13	0, 71 – 6, 39	
ATV/r	20 (38, 5)	33 (30, 8)	2, 64	0, 88–7, 96	
DRV/r	3 (5, 8)	18 (16, 8)	1, 00	–	
FPV/r	5 (9, 6)	1, (0, 9)	5, 83	1, 93 – 17, 65	
ART rescue with RAL					0, 050^a^
YES	5 (9, 6)	24 (22, 4)	1, 00	–	
NO	47 (90, 4)	83 (77, 6)	2, 10	0, 91 – 4, 81	
GSS					0, 947^a^
< 2	8 (15, 4)	15 (14, 0)	1, 11	0, 57 – 2, 14	
2	22 (42, 3)	44 (41, 1)	1, 06	0, 65 – 1, 72	
> 2	22 (42, 3)	48 (44, 9)	1, 00	–	

^a^Chi-square test ^b^Fisher’s exact test; *PR* prevalence ratio, *CI* Confidence interval, *NNRTI* nonnucleoside reverse transcriptase inhibitors, *NRTI* nucleoside/nucleotide reverse transcriptase inhibitors, *AZT* zidovudine, *TAM* analogous thymidine mutation, *3TC* lamivudine, *TDF* tenofovir, *PI* protease inhibitor, *LPV/r* lopinavir/ritonavir, *ATV/r*- atazanavir/ritonavir, *DRv/r* Darunavir/ritonavir, *FPV/r* fosamprenavir/ritonavir, *GSS* genotypic sensitivity score

The number of NRTI(t)-associated mutations did not affect virologic suppression (9.3% for zero NRTI(t)-associated mutations vs 48.6% for 1–2 NRTI(t)-associated mutations vs 42.1% for ≥3 NRTI(t)-associated mutations, *p* = 0.179) (Table 5).

**Table 5:** Genotypic and immuno-trophic characteristics at the time of genotyping associated with virological suppression after 48 weeks of initiation of the rescue scheme

Viral Load (VL)					
Characteristics	Detectable *n* (%)	Undetectable *n* (%)	PR	PR 95% IC	*P*-value
Age (years)					0, 662^a^
18–30	6 (11, 5)	13 (12, 2)	0, 92	0, 45 – 1, 86	
31–50	40 (76, 9)	76 (71, 0)	1, 00	–	
> 50	6 (11, 5)	18 (16, 8)	0, 73	0, 35 – 1, 52	
Gender					0, 385^a^
Male	36 (69, 2)	81 (75, 7)	0, 81	0, 50 – 1, 29	
Female	16 (30, 8)	26 (24, 3)	1, 00	–	
VL on genotypic result (copies/ml)					0, 752^a^
≤ 10.000	18 (35, 3)	36 (34, 3)	1, 00	–	
> 10.000 a 100.000	20 (39, 2)	47 (44, 7)	0, 90	0, 53 – 1, 52	
> 100.000	13 (25, 5)	22 (21, 0)	1, 11	0, 63 – 1, 98	
CD4 on genotypic result (cells/mm3)					0, 796^a^
< 200	20 (38, 5)	45 (42, 9)	0, 95	0, 54 – 1, 66	
200–350	18 (34, 6)	31 (29, 5)	1, 13	0, 64 – 1, 99	
> 350	14 (26, 9)	29 (27, 6)	1, 00	–	
Number of NRTI resistance mutations					0, 179^a^
zero	6 (11, 6)	10 (9, 3)	1, 00	–	
< 3	32 (61, 5)	52 (48, 6)	1, 02	0, 51 – 2, 02	
≥ 3	14 (26, 9)	45 (42, 1)	0, 63	0, 29 – 1, 38	
N° of thymidine analogue mutation					0, 058^a^
zero	35 (67, 3)	54 (50, 4)	1, 00	–	
< 3	14 (26, 9)	34 (31, 8)	0, 74	0, 45 – 1, 24	
≥ 3	3 (5, 8)	19 (17, 8)	0, 35	0, 12 – 1, 02	
Number of NNRTI resistance mutations					0, 113^a^
≤ 2	32 (61, 5)	79 (73, 8)	1, 00	–	
> 2	20 (38, 5)	28 (26, 2)	1, 45	0,93 – 2,25	
Number of PI resistance mutations					1, 000^b^
≤ 2	4 (80, 0)	14 (77, 8)	1, 00	–	
> 2	1 (20, 0)	4 (22, 2)	0, 90	0, 13 – 6, 35	
M184 V/I mutation					0, 693^a^
Yes	44 (84, 6)	93 (86, 9)	1, 00	–	
no	8 (15, 4)	14 (13, 1)	1, 13	0, 62 – 2, 07	

^a^Chi-square test ^b^Fisher’s exact test, *PR* prevalence ratio, *CI* Confidence interval, *CD4: CD4 T* cells, *CV* viral load, *NNRTI* non-nucleoside reverse transcriptase inhibitor, *PI* protease inhibitor, *NRTI* nucleoside reverse transcriptase inhibitor

After an analysis with the Poisson regression model, only being on ARVs for more than 36 months until genotyping was a protective factor for a detectable viral load (PR 0.6, 95% CI = 0.39–0.92, p = 0.02) 48 weeks after switching to the salvage regimen (Table 6).

**Table 6:** Poisson model for viral load detectable after 48 weeks of onset of rescue scheme

Variables	PR^a^	PR IC95%^b^	*p*-value
^c^∆t on ARV (months)			
≤ 36	1,00	–	0,020
> 36	0,60	0,39 – 0,92	

^a^PR: prevalence ratio ^b^IC: Confidence interval ^c^time variation on antiretroviral therapy

For the 153 patients with documented CD4 after 48 weeks, the median was 376 cells/mm3 (Q1 246; Q3 553) and the median CD4 gain was 125 cells/mm3 (Q1 47; Q3 243). In the population with virologic success, the variation in the CD4 gain above 100 cells/mm3 was significant when the VL at the time of genotyping was 10,000–100,000 copies/ml (69.8% vs 30.2% for variation < 100 cells/mm3, *p* = 0.047) and when the CD4 at the time of genotyping was below 200 cells/mm3 (81.4% vs 18.6% for CD4 < 100 cells/mm3 *p* = 0.010).

## Discussion

After evaluating 184 genotyping tests from patients during the first virologic failure, we found a higher prevalence of subtype B, of the M184 V/I and K103 N mutations, as well as a high frequency of NRTI(t) and NNRTI-associated mutations, with no impact on virologic suppression. We observed that the salvage therapy regimen was predominantly composed of PI/r and NRTI(t)s, with virologic success in most cases. Subtype B remains the most common in Pernambuco [17–19] and in Brazil [11], except in the south, where subtype C [21] is predominant. There has been an increase in the proportion of recombinant forms in Rio de Janeiro [22] and subtype F in Minas Gerais [40].

The elevated presence of M184 codon mutations is expected and arises as a consequence of the use of lamivudine as part of all the first-line regimens in our study. This drug confers a high level of resistance to cytosine analogs (lamivudine and emtricitabine), a low level of resistance to abacavir, and the increased susceptibility of zidovudine and TDF. In addition, it decreases the replication capacity of HIV-1 [23, 24]. Its presence has been associated with virologic success [10], but we did not observe this success in the present study.

Similar to our results, the high prevalence of M184 V/I mutations was reported in several regions of Brazil [11, 25, 40], in Sub-Saharan Africa [26] and in Asia [27], but to a lesser extent in western Europe [28]. This difference can be explained by the use of emtricitabine in European countries and by the use of lamivudine in low- and middle-income settings. However, in a recent meta-analysis [29], lamivudine and emtricitabine were clinically equivalent. All the genotype sequences of the non-B subtype (F and BF) had the M184 V/I mutation, probably due to the high prevalence of this mutation and the very low frequency of non-B subtypes in our study.

We found no association between the number of NRTI(t)-associated mutations and the ARVs used at the time of genotyping, including ARV regimens with or without TDF. There are studies showing a greater number of resistance-associated mutations among AZT [30] and TDF users [10, 31]. However, those studies had populations with different characteristics, especially with regard to subtype prevalence. A higher number of mutations with a TDF-based regimen was reported for both the B subtype^10^ as well as with the C subtype [33]. The association between a high number of NRTI(t)-associated mutations with CD4 pre-ARV and at the time of genotyping of > 200 cells/mm3 suggests a late diagnosis of PLHIV in our population, with an impact on the genotypic profile. These factors did not remain significant after the analysis with the Poisson regression model.

The K103 N/S mutation was documented in most genotypic sequences, due to the frequent use of EFV as the primary NNRTI in the first-line therapy for more than a decade in Brazil. These findings are similar to the national data [10–12] and data from low- and middle-income countries [14, 15, 26, 27]. The susceptibility of etravirine, a second-generation NNRTI, was not influenced by experiencing a long period of time in virologic failure while using EFV or NVP, or by the number of NNRTI-associated mutations.

This analysis was hampered by the small number of NVP users at the time of virologic failure. However, some studies [32–34] have shown that NVP-containing ARVs have repercussions on the response to etravirine for selecting the Y181C and G190A mutations, whereas the K103 mutation associated with EFV does not interfere with etravirine susceptibility.

The median time from ARV therapy to genotyping was similar to that of recent studies in Brazil [10], 10 Africa and Europe [36]. After the Poisson regression model analysis, remaining on ARV for more than 36 months until genotyping was the only factor that impacted the virologic response 48 weeks after the onset of salvage therapy, appearing as a protective factor for a detectable viral load. To understand the impact of time on ARV on virologic success, factors that could influence treatment adherence would have to be identified and controlled, because the major cause of acquired virologic failure is poor adherence to ARVs [42].

A shorter time on ARV is used as the best scenario for adherence in the calculation of odds ratio in studies that seek to understand the risk factors for treatment adherence. However, after association testing, its impact on adherence is not always confirmed [43]. This finding is probably observed because adherence is a dynamic behavior and varies over time for the same individual, and it is affected by diverse factors such as psychosocial-, ARV- and clinical scenario-related factors. This finding explains why the time on ARV may be a risk or protective factor for poor adherence, with an impact on virologic success [42].

By contrast, more than 36 months on ARV increased the risk for the selection of three or more NRTI(t)-associated mutations, although a longer time in virologic failure did not generate the same outcome. According to the virologic failure criterion of this study, individuals with low VL (> <1000 copies/ml) were not considered to be in virologic failure, because the genotyping tests available in Brazil during the data collection period were not able to expand the genetic material when the VL was < 1000 copies/ml. However, there is evidence that having a VL of > 200 copies/ml increases the risk for virologic failure and may lead to mutation accumulation [44]. Therefore, it is possible that many individuals who are on ARV for a long period of time (> 36 months) had low VL but were not diagnosed with virologic failure, accumulating mutations.

We observed that the presence of NRTI(t)-associated mutations or TAMs at the first virologic failure did not interfere with virologic suppression 48 weeks after the switch to a second-line therapy with NRTI(t)s combined with PI/r. This finding is not only due to the high potency of PI/r or to the direct activity of NRTI(t)s, and it may reflect an effect of the NRTI(t)s on viral fitness [35]. This finding is consistent with the results of three large randomized controlled trials in which patients with extensive resistance to reverse transcriptase evolved with virologic success after salvage therapy based on NRTI(t) with PI/r [13–15].

We found no difference in the virologic response between regimens with an individual GSS of less than two and those greater than or equal to two, confirming the hypothesis that there is no difference between active, partially active or inactive NRTI(t)s in terms of virologic success [35]. In the EARNEST study [14], the group receiving inactive or partially active NRTI(t)s exhibited viral load suppression that was similar to if not better than that exhibited by the group receiving the regimen containing a fully active drug from a new class, and it was superior to that exhibited by the group using only a protease inhibitor.

It is possible that pharmacokinetic characteristics optimize the benefits of NRTI(t)s even if inactive. Thus, ARVs with a long intracellular half-life, such as TDF and lamivudine, may help the PI/r to maintain viral suppression. This effect is independent of the drug activity [41].

We were able to define the time in virologic failure until the switch to second-line therapy. Surprisingly, most of the patients with undetected viral loads were in virologic failure for more than 12 months. Because the time under virologic failure did not interfere with the number of NRTI(t)-associated mutations or TAMs in our study, we can suggest that the resistance-associated mutations arose during the first months of virologic failure. In the EUROSIDA study [37], a high number of TAM-1 mutations was observed within one year of failure, with a lower accumulation rate than what was predicted among those who stayed on the failing regimen.

The total absence of ARV selective pressure during part of the virologic failure period is another way to explain why the longer virologic failure time did not influence the number of NRTI(t)-associated mutations or TAMs. The complete withdrawal of ARVs generates fewer resistance-associated mutations than does the maintenance of sub-therapeutic doses [9]. It was not possible to determine whether the study participants were kept on sub-therapeutic doses or if they completely abandoned the treatment when they were in virologic failure.

When we analyzed the virologic response and ARVs used in salvage therapy, we observed that there was no difference between the patients who used AZT or TDF or both. The use of LPV/r was significant among those who experienced virologic success, probably because it was the most frequently prescribed PI during the study period, as directed by the Brazilian Ministry of Health. Among users of DRV/r or RAL, the majority achieved virologic success, although they were smaller in absolute numbers.

DRV/r is the newest PI and has a high genetic barrier, in addition to fewer adverse effects than LPV/r, possessing activity even with a protease mutation [38]. When the protease is not intact, adding a new ARV class is critical. This class has often been the integrase inhibitor [39]. It should be noted that this study was not designed to assess the individual power of each regimen. Among those who reached an undetectable VL, a CD4 gain above 100 cells/mm3 was significant in subjects with CD4 at the time of genotyping > 200 cells/mm3, demonstrating that when there is adherence, a significant immunologic gain is possible even in patients with a low immunologic reserve and extensive resistance in reverse transcriptase.

Our primary limitation was the difficulty in establishing cause-and-effect relationships, because this was a cross-sectional study. Our results may have been partially affected by data we were unable to collect, particularly those related to adherence to ARV therapy, and by the loss of information in some variables. However, this study has relevant points because it updates the genotypic data of an important region of Brazil with limited resources. In addition, we were able to establish the time in virologic failure and the ARV regimen before and after genotyping in the first virologic failure as well as the factors associated with the number of NRTI(t)-associated mutations and virologic success.

## Conclusions

Patients in their first virologic failure who were seen at referral centers in the city of Recife, Pernambuco, Northeastern Brazil presented a high frequency of mutations pertaining to secondary resistance to the use of NRTI(t)s and NNRTIs. However, extensive resistance in reverse transcriptase did not impact the second-line virologic suppression after 48 weeks of the salvage therapy with PI/r combined with active, partially active or inactive NRTI(t)s. The time on ARVs to genotyping was an independent protective factor for a detectable viral load. Long-term follow-up is needed to support the use of partially active NRTI(t)s in salvage therapy regimens while controlling for adherence-related factors. Studies assessing the actual need for genotyping after the first virologic failure with NNRTIs are also needed.

## Abbreviations

3TC: Lamivudine
ARVs: Antiretrovirals
ATV: Atazanavir
AZT: Zidovudine
CD4: CD4 T-cell counts
DRV/r: Darunavir/ritonavir
EFV: Efavirenz
GSS: Genotypic sensitivity score
IAS: International AIDS Society
LPV/r: Lopinavir/ritonavir
NNRTI: Non-nucleoside reverse transcriptase inhibitor
NRTI(t): Nucleos(t)ide reverse transcriptase inhibitor
NVP: Nevirapine
PI: Protease inhibitors
PLHIV: People are living with HIV
SPSS: Statistical package for the social sciences
TAMs: Thymidine-associated mutations
TDF: Tenofovir
VL: Viral load
